# How Adalimumab Impacts Antibiotic Prescriptions in Patients Affected by Hidradenitis Suppurativa: A 1-Year Prospective Study and Retrospective Analysis

**DOI:** 10.3390/jcm12030837

**Published:** 2023-01-20

**Authors:** Fabrizio Martora, Claudio Marasca, Vincenzo Picone, Luigi Fornaro, Matteo Megna, Gabriella Fabbrocini

**Affiliations:** 1 Section of Dermatology, Department of Clinical Medicine and Surgery, University of Naples Federico II, 80138 Napoli, Italy; 2 Melanoma, Cancer Immunotherapy and Development Therapeutics Unit, Istituto Nazionale Tumori IRCCS Fondazione “G. Pascale”, 80131 Napoli, Italy

**Keywords:** hidradenitis suppurativa, DLQI, VAS pain, IHS4, adalimumab, treatment

## Abstract

We conducted a one-year prospective study involving the enrollment of 58 patients with Hidradenitis Suppurativa. Through a retrospective analysis of data on the same patients, with reference to the year prior to the initiation of the anti-TNFα drug adalimumab, we aimed to show how the advent of this biologic therapy changes the number of days of antibiotic therapy, the number of flare-ups per year, and their duration in days, as well as the quality of life and perceived pain of patients.

## 1. Introduction

Hidradenitis Suppurativa (HS) is a chronic, relapsing, inflammatory skin condition. It is characterized by the occurrence of nodules, which are often inflammatory, deep and painful, especially in skin regions with a high density of apocrine sweat glands such as the axillary, inguinal, and sub mammary regions [1].

The exact prevalence of HS is not known. European studies have shown that it is a condition that is far from rare [1]. It is considered to have an overall prevalence ranging from 1 to 4.1 percent, with a female: male ratio of 3.3:1 [2]. The onset of manifestations typically occurs between the second and third decades, and the average age of first onset is 21 years [1,2].

HS is associated with several other pathological conditions. These include obesity, metabolic syndrome, and autoimmune disorders such as Crohn’s disease [3]. Patients suffer as much physically as psychologically, with worsening quality of life and a decline in work productivity. The numerous comorbidities make the management of the patient with HS extremely complex, with the need for a multidisciplinary approach to ensure the best chance of therapeutic management [4].

The application of staging and classification systems in the management of patients with HS arises from the need to produce treatment plans that are appropriate to the individual case and that consider the severity of the specific lesions. The two main tools are represented by Hurley’s and Sartorius’ systems, the former of which is qualitative and the latter of which is quantitative. They are complemented by other systems such as the PGA (HS-Physician’s Global Assessment), the HSSI (HS Severity Index), and the AISI (Acne Inversa Severity Index). Additional scoring systems include the DLQI (Dermatology Life Quality Index), which is necessary to investigate the patient’s quality of life, and the VAS (Visual Analogue Scale), which is used to quantify the patient’s perceived pain. The presence of multiple systems is necessary because none of them turns out to be completely sufficient to assess the severity of the pathology, and it is possible to identify the relative advantages and disadvantages of each [5,6,7].

Another parameter commonly used to classify lesions is high-frequency ultrasound; very often the clinical parameter does not match the ultrasound parameter because the latter has a higher sensitivity than the clinical evaluation, so it becomes essential to use ultrasound to determine the severity of the lesions [8,9].

To date, antibiotics and biologics are the most widely used treatments for the disease. On the one hand, antibiotics are useful in improving the severity of the disease but particularly in managing over-infections; unfortunately, they do not have long-term efficacy because they can be used for short periods of time; on the other hand, adalimumab is the only biologic drug approved for HS. The combination of these two therapies has led to good and effective therapeutic responses in real-life experiences [10].

## 2. Methods

We conducted a one-year prospective study involving the enrollment of 58 patients with HS.

### 2.1. Primary Endpoint

Through a retrospective analysis of data on the same patients, with reference to the year prior to the initiation of the anti-TNFα drug adalimumab, we aimed to show how the advent of this biologic therapy changes the number of days of antibiotic therapy, the number of flare-ups per year, and their duration in days, as well as the quality of life and perceived pain of patients.

### 2.2. Secondary Endpoint

Quality of life was assessed using the DLQI. Patient-perceived pain was quantified through the VAS scale. Disease severity was assessed clinically using the International Hidradenitis Suppurativa Severity Score System (HIS4) clinical score [11,12,13].

The collected data were processed using GraphPad Prism software (GraphPad Inc., La Jolla, CA, USA). Parameters were calculated for each variable (number of days of antibiotic therapy given, number of annual flare-ups, duration of flare-up, IHS4, VAS, DLQI) using mean ± standard deviation. The paired *t*-test was used to compare the data collected before starting adalimumab and after 1 year of therapy. The same test was used to compare data from the year before starting adalimumab treatment.

## 3. Results

Listed below are the results obtained from the analysis of the variables studied in the sample, consisting of 58 patients (27 males and 31 females, mean age 32.58 years) with HS. The patients received the same adalimumab therapy for one year: induction phase: 160 mg (4 × 40 mg or 2 × 80 mg) at T0, 80 mg (2 × 40 mg or 80 mg) after 2 weeks, and then 40 mg after another 2 weeks; maintenance phase: 40 mg/week [14].

First, we evaluated the number of days for which antibiotics were used by patients during treatment with adalimumab. At T1, after 12 months of treatment, patients had performed an average of 21.48 ± 20.47 days of antibiotic therapy. From the recorded data, it was found that in 58% of the cases, the oral antibiotic combination of clindamycin (600 mg/day) and rifampin (600 mg/day) was prescribed; in 30% of the cases, there was the use of limecycline 300 mg; and in the remaining 12%, there was the use of doxycycline 100 mg for two times/day.

Regarding disease flare-ups, we analyzed two variables, namely the number of flare-ups and their duration in days. We obtained 2.32 ± 2.62 annual flare-ups at T1, and their duration averaged 3.90 ± 1.34 days.

### 3.1. Primary Endpoint

**Figure 1:**
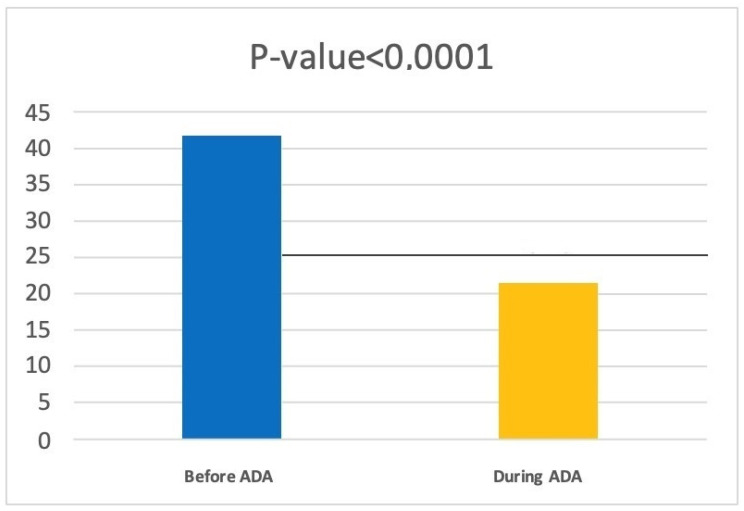
Data regarding the number of antibiotic therapy days before and during adalimumab.

Through a retrospective analysis conducted on the same patients, we traced the number of days of antibiotic therapy they were taking in the year prior to the start of adalimumab treatment and found an average of 41.47 ± 26.43 days of antibiotic therapy. In a comparison of the two averages through a paired *t*-test, we noted that there was a reduction of 20.28 days of antibiotic therapy (*p* < 0.0001) (Figure 1).

Regarding disease flares, we analyzed two variables, namely the number of flare-ups and their duration in days. Then, these data were also compared through a retrospective analysis conducted on the same patients before they started treatment with adalimumab. We found a reduction in the number of flare-ups: before adalimumab, patients had an average of 4.20 ± 3.03 flare-ups per year, whereas at T1, we recorded 2.32 ± 2.62 flare-ups per year. The duration of flares was also remarkably reduced from an average of 5.94 ± 0.89 days before starting anti-TNFα to an average of 3.90 ± 1.34 days at T1.

### 3.2. IHS4 Score

**Figure 2:**
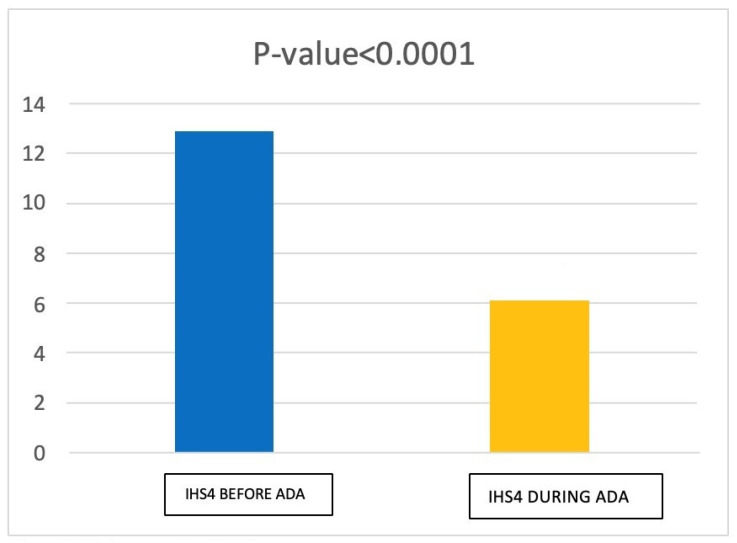
Data regarding IHS4 before and during treatment with Adalimumab.

Regarding International Hidradenitis Suppurativa Severity Score Syhism (IHS4), at T1 we found a mean score of 6.10 ± 3.66 (moderate grade HS). Prior to the initiation of adalimumab therapy (T0), we found that patients had an average IHS4 score of 12.88 ± 4.05 (severe grade HS). Thus, we obtained a statistically significant reduction in IHS4 score at T1 (*p* < 0.0001). (Figure 2).

### 3.3. DLQI

**Figure 3:**
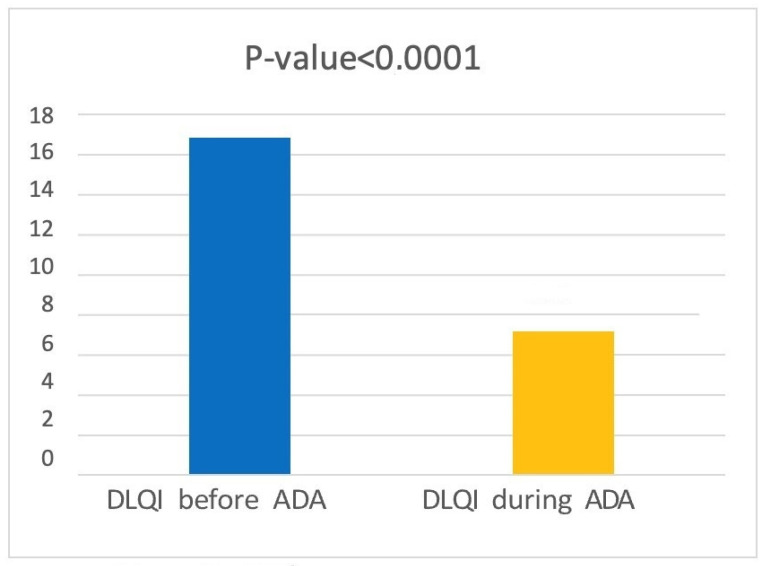
Data regarding DLQI before and during treatment with Adalimumab.

The DLQI (DERMATOLOGY LIFE QUALITY INDEX) was assessed before the start of adalimumab treatment and at T1. We found an average score of 7.16 ± 4.42 at T1 (disease mildly or moderately affects patient’s quality of life), whereas before starting treatment, the average score was 16.84 ± 4.73 (disease greatly or extremely affects patient’s quality of life). A *t*-test showed a statistically significant reduction in DLQI score (*p* < 0.0001) (Figure 3).

### 3.4. VAS Pain

**Figure 4:**
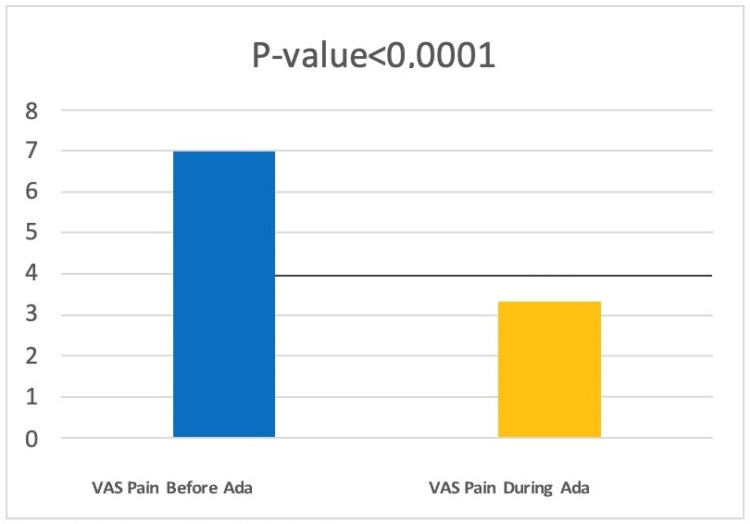
Data regarding Vas Pain Score before and during treatment with Adalimumab.

Finally, we evaluated how adalimumab therapy can change the VAS pain scale score. Patients at T1 had an average score of 3.32 ± 1.65. Comparing this to the scores obtained before the start of treatment (6.98 ± 1.30), through a paired *t*-test, we found a statistically significant reduction in the VAS scale score for pain (*p* < 0.0001) (Figure 4).

## 4. Discussion

HS is a skin condition of which clinical management has always been a challenge for dermatologists. In fact, the therapies currently available allow mostly partial benefits with significant implications for the quality of life of affected patients. Antibiotic therapy has always been one of the first choices in the treatment of this condition [15].

The main objective of this study was to evaluate whether patients with HS being treated with adalimumab present a benefit in terms of reduced antibiotic therapy prescriptions. Adalimumab is approved for the treatment of patients with moderate–severe HS resistant to conventional therapies, such as antibiotics [15].

In general, in clinical practice, antibiotics are mainly prescribed during periods of exacerbations of the disease. Therefore, it is natural to hypothesize that in HS patients on adalimumab therapy, as the flare-ups are reduced, the antibiotics prescribed will be reduced. In fact, in our study, adalimumab was shown to reduce both the number of annual flare-ups and their duration in days, as well as the number of antibiotic prescriptions. What emerges from the current studies is that antibiotic therapy remains a valid therapy in clinical practice to manage disease flares, but at the same time, there is evidence of their over-prescription [16,17].

Evaluation on discontinuation or continuation of antibiotic therapy during the study was clinical by referring to the disease flare-ups and scores used.

In fact, Kitts S. et al. showed that out of 9293 people with HS, 52.9% had antibiotic therapy. Among them, 82.9% of patients received antibiotic treatment lasting ≥30 days, and the average duration was 12 weeks. Such prolonged antibiotic therapies only increase antibiotic resistance, which is also a cause of prolonged antibiotic therapy [18,19].

To improve acute symptomatology in patients with few lesions, good results are also reported with the use of intra-lesional corticosteroids [5]. In fact, in several case reports [20], statistically significant results were found in terms of improvements in clinical response and quality of life after administration of intralesional triamcinolone acetonide (ILTAC) at high doses (20–40 mg/mL). The best results were obtained when the disease had single-location and mild-to-moderate manifestations. However, the authors admitted that ILTAC treatment could be ineffective or clinically impractical in cases of multiple manifestations [20]. The authors hypothesized that there is a synergism between corticosteroids (administered both intralesionally and systemically) and adalimumab [20]. The mechanism of action of corticosteroids in the treatment of HS is blocking the synthesis of leukotrienes and of proinflammatory cytokines through activation of the receptors of the glucocorticosteroids, and this could have a synergistic effect with the treatment with anti-TNFα [19,20,21]. In conclusion, another parameter that needs to be considered regarding the response or nonresponse to treatment and, consequently, the reduction in the number of days of antibiotic therapy undertaken and the number of flare-ups are certainly the risk factors; these certainly go into this assessment as widely described in the literature [12,13,14,15,16,17,18,19,20,21].

## 5. Conclusions

Currently, HS represents an ongoing therapeutic challenge for dermatologists, who are often faced with complex patients with multiple comorbidities who do not respond to currently available therapies [21]. For this reason, it is necessary to reconsider this pathology from a multidisciplinary point of view in which biologic therapy finds its role not only in reducing antibiotic prescriptions but also in giving the patient a stability of the inflammatory state that makes it possible to reduce the flare-ups of the pathology. Biologic therapy with adalimumab has been the subject of several studies in the literature, and it is now agreed that it represents a valuable tool, as its efficacy and safety have been confirmed, as well as its positive influence on patients’ quality of life [22,23].

However, it should be kept in mind that a variable proportion of patients on adalimumab therapy experience flare-ups, more or less frequently. The therapeutic approaches that are available, including antibiotic therapy, to manage flare-ups are varied, and we believe that their combined, integrated, and targeted use is necessary to achieve disease stability [24].

Due to the lack of effective treatments for moderate to severe HS, new therapeutic options are being investigated that target specific cytokines involved in HS pathogenesis [25]. Inhibition of anti-IL-17 and anti-IL-1α appear to be promising therapeutic options for moderate to severe HS that is refractory to adalimumab. In this regard, new phase III studies are underway in which anti-interleukin (IL)-17 biologic drugs, including bimekizumab and secukinumab, are showing promising results, but further studies are certainly needed to confirm their efficacy [26,27,28].

It is hoped that current and future investigations of HS biomarkers will lead to a better assessment of the disease and that further understanding of the inflammatory phenomena involved and cytokine cascades will lead to highly effective precision targeted therapy. In conclusion, our study showed that adalimumab therapy dramatically reduced the number of antibiotic therapy prescriptions while reducing the number and duration of flare-ups. The bias of our study may be due to the retrospective aspect of antibiotic prescriptions vs. the prospective aspect of adalimumab, so we believe that further studies are needed to confirm our results.

## Data Availability

Data are reported in the current study and are available on request from the corresponding author.
